# Draft genome sequence of multidrug-resistant *Enterobacter hormaechei* F10K from a captive common peafowl (*Pavo cristatus*) with respiratory illness and enteritis

**DOI:** 10.1128/mra.01029-25

**Published:** 2025-11-10

**Authors:** Abu Nasar Md. Aminoor Rahman, Khaled Hassan Shuvo, Tima Tisa Mallick, Naim Siddique, Md. Arman Sharif, M. Nazmul Hoque

**Affiliations:** 1 Molecular Biology and Bioinformatics Laboratory, Department of Gynecology, Obstetrics and Reproductive Health, Gazipur Agricultural University 198780, Gazipur, Bangladesh; 2 Department of Anatomy and Histology, Gazipur Agricultural University 198780, Gazipur, Bangladesh; University of Maryland School of Medicine, Baltimore, Maryland, USA

**Keywords:** draft genome, *E. hormaechei*, multidrug resistance, virulence factors, peafowl

## Abstract

We report the draft genome sequence of *Enterobacter hormaechei* strain F10K, a multidrug-resistant bacterium isolated from the feces of a captive common peafowl. The assembled genome is approximately 4.63 Mbp in size, with a guanine–cytosine (GC) content of 55.5% and an estimated completeness of 99.27%.

## ANNOUNCEMENT

*Enterobacter hormaechei*, a gram-negative bacterium of the *Enterobacter cloacae* complex (ECC) (1). It is an opportunistic pathogen that affects immunocompromised hosts (2, 3). Although uncommon in animals (4), *E. hormaechei* has recently been identified as a multidrug-resistant ECC species causing various infections in pigs, cattle, sheep, and fox (5–9).

Freshly voided fecal samples were aseptically collected from a 2-year-old common peafowl diagnosed to show respiratory distress with open-mouth breathing and whitish diarrhea before death. The bird was housed at the Wildlife Reproduction and Conservation Field Laboratory, Gazipur Agricultural University (24.09° N, 90.41° E), Bangladesh. Samples were collected using sterile stool tubes, kept at 4°C in an icebox, and transported to the lab for processing within 24 h. Feces samples were diluted with sterile distilled water, vortexed, and preserved for further analysis. For *E. hormaechei* isolation, samples were enriched overnight in nutrient broth (Oxoid, UK) at 37°C under aerobic conditions, serially diluted (10⁻³), and streaked onto MacConkey agar (Oxoid). After 24  h of incubation, distinct colonies were subcultured to obtain pure isolates. The bacterium was identified as *E. hormaechei* based on colony morphology, Gram staining (gram-negative rods, pink colonies), and biochemical tests, including the Methyl Red-Voges-Proskauer (MR-VP) test (10, 11). Species-level identification of *E. hormaechei* was confirmed using the VITEK-2 system v.9.01 (12). The F10K isolate showed resistance to ampicillin, aztreonam, cefoxitin, chloramphenicol, ciprofloxacin, nalidixic acid, nitrofurantoin, oxacillin, and tetracycline, as determined by Kirby-Bauer disk diffusion following Clinical and Laboratory Standards Institute M100 (2023) guidelines (13). For DNA extraction, strain F10K was grown in nutrient broth at 37°C for 18–24 h under aerobic conditions. Genomic DNA was extracted with the QIAamp DNA Mini Kit (QIAGEN, Germany) and quantified using NanoDrop 2000 (Thermo Fisher, USA) (14, 15). Whole genome sequencing libraries were constructed from 1 ng of DNA using the Nextera DNA Flex Kit (Illumina, USA) following the manufacturer’s instructions. The process involved enzymatic fragmentation and size selection, and sequencing was performed on an Illumina MiSeq platform using a 2 × 250 bp paired-end protocol. Raw reads (*n* = 4,244,402) were trimmed with Trimmomatic v.0.39 (16) and assessed with FastQC v.0.12.1 (17).

Genome assembly was performed with SPAdes v.4.2.0 (18) and annotated using the National Center for Biotechnology Information Prokaryotic Genome Annotation Pipeline v.6.10 (19). Genome completeness, virulence factor genes (VFGs), antibiotic resistance genes (ARGs), metabolic pathways, and pathogenic potential were evaluated using CheckM v.1.2.3 (20), VFDB v.6.0 (21), ResFinder 4.0 (22), RAST server (23), and PathogenFinder2 v.0.5.0 (24), respectively, with default parameters for all software unless otherwise specified.

The key genomic features of *E. hormaechei* F10K are shown in Table 1. We identified 22 ARGs, 21 VFGs, and 372 functional subsystems. The strain was associated with 105 pathogenic families by yielding a pathogenicity score of 0.966. These findings highlight *E. hormaechei* F10K as a potential pathogen associated with respiratory illness and enteritis in peafowls, emphasizing its relevance for veterinary and public health research.

**TABLE 1:** Genomic features of the *E. hormaechei* F10K isolated from the feces of a captive common peafowl with respiratory illness and enteritis^*a*^

Genomic features	*E. hormaechei* strain F10K
Genome size (bp)	4,632,952
Genome coverage (×)	112
Genome completeness (%)	99.27
CheckM contamination (%)	0.62
No. of contigs	50
Contig N50 (bp)	312,937
GC content (%)	55.5
Coding sequences (CDSs)	4,402
Protein-coding genes	4,358
No. of SEED subsystems	372
No. of VFGs	21
No. of ARGs	22
PathogenFinder2 score	0.966
Genome accessions	JBOEQC000000000
Sequence Read Archive accessions	SRR33697069

^*a*^ SEED, Subsystems Enabling the Effective Determination of function.

## Data Availability

The whole-genome shotgun sequencing project of *Enterobacter hormaechei* strain F10K has been deposited in GenBank and the National Center for Biotechnology Information Sequence Read Archive (SRA) under BioProject accession number PRJNA1267841. The SRA accession is listed in Table 1. The version described in this paper is version JBOEQC000000000.1.
